# Analysis of Rhesus (Rh) Antigen Distributions in Donors and Multi-transfused Patients for Phenotype-Matched Transfusion

**DOI:** 10.1007/s12288-023-01676-9

**Published:** 2023-07-08

**Authors:** Yuhong Zhao, Ni Yao, Yan Lv, Dawei Cui, Jue Xie

**Affiliations:** Department of Blood Transfusion, The First Affiliated Hospital, Zhejiang University School of Medicine, Hangzhou, 310003 China

**Keywords:** Rh phenotype-matched transfusion, Alloimmunization, Safety of blood transfusion, Rh antigen distribution

## Abstract

Knowledge about the frequency of Rh blood group systems in the local population help build a donor pool for multi-transfused patients and provide antigen-negative compatible blood for patients with alloantibodies. ABO and Rh antigens were identified for blood donors and patients before transfusion. The antiglobulin test based on the micro-column gel method was used to perform unexpected antibody screening and identification for patients in pre-transfusion testing. The incidence of the adverse transfusion reactions and the accordance rate of Rh phenotype-matched transfusion were analyzed retrospectively. A total of 246,340 specimens were detected with Rh blood group antigens D, C, E, c, and e. Rh D antigen was the most common phenotype with a frequency of 99.40%, followed by e antigen, C antigen, c antigen, and E antigen. In Rh D positive specimens, DCe was the most common phenotype, while DCE was the least common. At the same time, in Rh D negative specimens, ce was the most common phenotype with CE and CcE unobserved. Rh phenotype-matched transfusion has been conducted in our department since 2012. The accordance rate of Rh phenotype-matched transfusion has been kept above 95% and the resulting incidence of adverse transfusion reactions has been decreasing year by year, from 19.95‰ in 2011 to 2.21‰ in 2021. Blood transfusion with matched Rh phenotypes was able to avoid the generation of unexpected antibodies, reduce the incidence of adverse transfusion reactions, and enhance precise diagnosis and treatment.

## Introduction

Since the 1940s, many blood group antigens have been discovered. Till now, 43 blood group systems have been identified with 345 red blood cell (RBC) antigens [1, 2], according to the International Society for Blood Transfusion (ISBT). Among these blood group systems, the Rh blood group system has been the most polymorphic with 55 antigens confirmed by the ISBT. Of these antigens, Rh antigens D, C, E, c, and e are the most clinically relevant with their antigenicities from the strongest to the weakest being D > E > C > c > e [3]. Currently, Rh D and ABO blood group systems have been recognized as the most significant clinically due to their massive applications in clinical blood transfusion therapy.

In most blood transfusion departments (blood banks) of various hospitals, only the matching of the ABO blood group system and Rh D blood group antigen are currently required between blood donors and patients in pretransfusion testing. However, random transfusion of ABO and Rh D compatible blood with unknown Rh phenotypes, especially in multi-transfused patients (*n* > 2), may lead to the generation of alloantibodies as a result of alloimmune responses. These alloantibodies are responsible for fatal hemolytic transfusion reactions (HTRs) and serious hemolytic disease of the fetus and the newborn (HDFN) [4–6].

Our department started to detect the other Rh-specific antigens (C, E, c, and e) in multi-transfused patients, be matched for the component blood transfusion of ABO and Rh phenotype since 2012, and generalized the detection to all patients who might need blood transfusion since 2014. Rh phenotype-matched transfusion can reduce the incidence of alloimmunization and the rate of adverse transfusion reactions for patients, especially in multi-transfused patients.

Detection of other Rh antigens (C, E, c, and e) in pretransfusion testing is beneficial to prevent alloimmunization, especially for multi-transfused patients [7–9]. To this end, our department started to detect Rh antigens C, E, c, and e in multi-transfused patients (*n* > 2), and conduct the component blood infusion with matched ABO and Rh phenotypes in 2012. Later in 2014, this kind of pretransfusion test was applied to all the patients in need of blood transfusion. After the Rh phenotype-matching transfusion, the incidence of adverse transfusion reactions in our hospital decreased year by year. In addition, the survival of RBCs was enhanced with the required number of transfusions decreased.

Here in this contribution, we would like to present the obtained results in terms of Rh antigens D C, E, c, and e in our hospital. Frequencies of Rh-specific antigens were calculated. The frequencies of Rh D phenotypes were determined and analyzed in Rh D positive specimens. The results were then compared with available data in the literature. The results showed that the crossmatch of Rh D phenotypes along with ABO for pre-transfusion compatibility testing is beneficial to the reduction of alloimmunization. In addition, the distribution of unexpected antibodies in patients was explored to provide clues for the generation of Rh blood group antibodies and related clinical solutions.

## Materials and Methods

### Studied Populations

In this study, 129,078 patient samples requiring clinical blood transfusion were collected in our hospital (The First Affiliated Hospital, College of Medicine, Zhejiang University) from January 2013 to August 2021 (A multi-transfused patient is regarded as one sample). 117,262 blood donor samples were provided by the Blood Center of Zhejiang Province (Duplicated samples were removed based on their Blood Center ID numbers). The study was approved by the Institutional Ethics Committee of our hospital, and written informed consent was obtained from all participants.

### Instruments and Reagents

For the antiglobulin test, micro-column gel reaction cards were purchased from DiaMed GmbH. The instruments and reagents for Rh blood group system identification were bought from Bioxun Biotech Co., Ltd. The panel cells (a set of 16 types) were purchased from Sanquin Reagents B.V. Ltd. The main instruments include an incubator (DiaMed GmbH.) and centrifuge (BASO Gas Products LLC).

### Methods

The Rh phenotypes of all donor and patient samples were detected according to the protocols of instrument providers. Antibodies were screened by antiglobulin test based on the micro-column gel method for all patients in need of clinical blood transfusion. The screened unexpected antibodies were then identified by saline method and antiglobulin test method. All the above laboratory test results were independently obtained by two different operators.

### Statistical Analysis

Frequencies of the Rh-specific antigens (D, C, E, c, and e) were obtained by direct counting of the number of given antigens. The final results were expressed as a percentage. Excel 2019 was used to manage and preprocess the data. All the statistical analysis was performed using the Rstudio software.

## Results and Discussion

A total of 246,340 samples were collected for the study, with 117,262 donor samples and 129,078 patient samples. Among these samples, 244,870 samples tested positive for Rh D antigen while the remaining 1470 samples tested negative. Both ABO and Rh antigens (D, C, E, c, and e) were detected for all 246,340 samples.

### Antigen and Allele Frequencies in the Total Population Studied

**Fig. 1:**
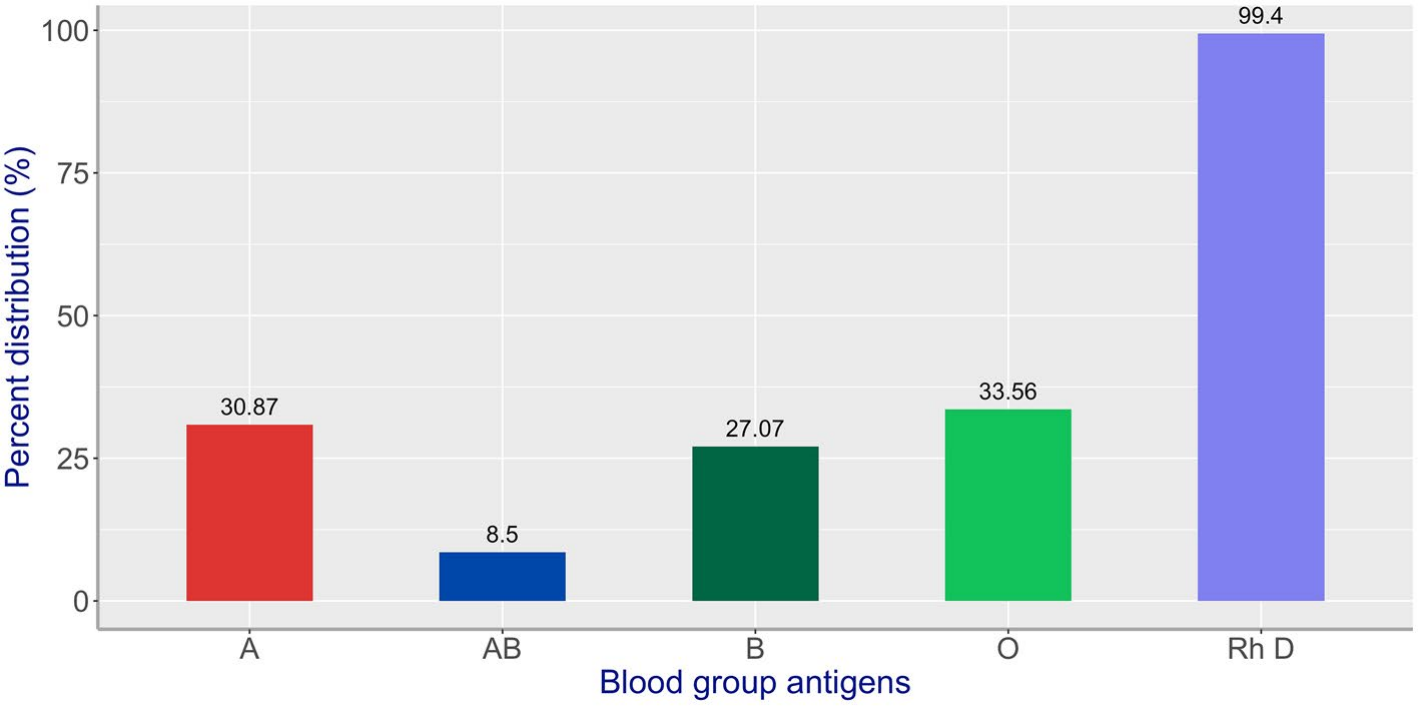
ABO and Rh D blood group distribution (*n* = 246,340)

The distributions of ABO and Rh blood group systems in all the patient and donor samples are shown in Fig. 1. The percent positive rate of Rh D in the total population studied was shown to be 99.40%. To be more accurate, in the patient population, the rates of positivity and negativity of Rh D were 99.6% (128,546 out of 129,078) and 0.4% (532 out of 129,078), respectively. Meanwhile, in the donor population, the corresponding rates of positivity and negativity were 99.2% (116,324 out of 117,262) and 0.8% (938 out of 117,262), respectively. As for the ABO blood group system, the most common blood group was found to be O, followed by A, B, and AB.

At the same time, the allele frequencies and corresponding prevalence of antigens for the Rh blood group system was shown in Table 1. A direct observation could be made that D (99.40%) was the most common Rh antigen, followed by e (92.61%), C (88.77%), c (53.63%), and E (44.94%).

**Table 1:** Rh antigens in the studied population (*n* = 246,340)

Antigen	Allele frequency in studied population (%)	Total number	Percentage prevalence of antigens	
			Present study (%)	95% CI**
D	–	244,870	99.40	[99.37, 99.43]
C	67.57	218,668	88.77	[88.64, 88.89]
E	26.17	110,712	44.94	[44.75, 45.14]
c	32.43	132,117	53.63	[53.43, 53.83]
e	73.83	228,141	92.61	[92.51, 92.72]

^**^95% confidence intervals

### Rh Antigen Frequencies in the Studied Population

We were also interested in the distribution of other Rh antigens (C, E, c, e) and their relations with the Rh D antigen. In this sense, we summarized the frequencies of different Rh antigens in the population that tested positive and negative for Rh D antigen in Tables 2 and 3, respectively.

**Table 2:** Prevalence of other Rh antigens in Rh D positive population (*n* = 244,870)

Antigen	ABO									
	A		B		O		AB		Total	
	Number	Percentage (%)	Number	Percentage (%)	Number	Percentage (%)	Number	Percentage (%)	Number	Percentage (%)
C	68,022	89.18	58,818	89.00	72,798	88.95	18,456	89.28	218,094	89.06
E	34,634	45.41	30,002	45.40	36,803	44.97	9179	44.40	110,618	45.17
c	40,968	53.71	35,453	56.65	43,527	53.19	10,802	52.25	130,750	53.40
e	70,631	92.61	61,215	92.63	75,656	92.44	19,174	92.75	226,676	92.57

**Table 3:** Prevalence of other Rh antigens in Rh D negative population (*n* = 1470)

Antigen	ABO									
	A		B		O		AB		Total	
	Number	Percentage (%)	Number	Percentage (%)	Number	Percentage (%)	Number	Percentage (%)	Number	Percentage (%)
C	181	39.01	120	35.82	207	41.57	66	38.15	574	39.05
E	15	3.23	27	8.06	42	8.43	10	5.78	94	6.39
c	425	91.59	312	93.13	477	95.78	153	88.44	1367	92.99
e	462	99.57	334	99.70	496	99.60	173	100.00	1465	99.66

As shown in Table 2, among the 244,870 specimens that tested positive for Rh D antigen, the most common Rh antigen was e (92.57%), followed by C (89.06%), c (53.40%), and E (45.17%). Hence, the frequencies of the Rh antigens were sequenced from the greatest to the smallest as D > e > C > c > E.

As for the 1470 specimens that tested negative for Rh D antigen, it was found that the most common Rh antigen was e (99.66%), followed by c (92.99%), C (39.05%), and E (6.39%). The resulting sequence of frequencies of Rh antigens was e > c > C > E, as shown in Table 3.

### Rh Phenotype Frequency

A more practical interest was placed upon the frequencies of Rh phenotypes in the population that tested positive for Rh D antigen. As shown in Table 4, nine Rh phenotypes were considered, in which the most common one was DCe (45.85%), followed by DCcEe (33.62%), DCce (8.61%), DcE (7.19%), and DcEe (3.338%). It should be noted that altogether 33 specimens showed the Rh phenotype DCE with a frequency smaller than 0.1%. In Table 4, we provided more information in the sense that the frequency distributions of Rh antigens were also tabulated with the related ABO blood group system. In this way, we were able to extract some information about the distribution of Rh antigens in the populations with different ABO blood groups. In this process, the 2019 version of the AABB standard for Rh phenotypes was adopted.

**Table 4:** Rh phenotypes frequency in the study of Rh D positive population (*n* = 244,870)

Antigen	ABO									
	A		B		O		AB		Total	
	Number	Percentage (%)	Number	Percentage (%)	Number	Percentage (%)	Number	Percentage (%)	Number	Percentage (%)
DCe	34,705	45.50	30,162	45.64	37,709	46.08	9693	46.89	112,269	45.85
DCEe	589	0.77	464	0.70	590	0.72	15	0.85	1818	0.74
DCE	9	0.01	8	0.01	14	0.02	2	0.01	33	0.01
DCce	6632	8.70	5697	8.62	7030	8.59	1725	8.34	21,084	8.61
DCcEe	25,915	33.98	22,339	33.80	27,266	33.32	6816	32.97	82,336	33.62
DCcE	172	0.23	148	0.23	189	0.23	45	0.22	554	0.23
Dce	300	0.39	226	0.34	298	0.36	75	0.36	899	0.37
DcEe	2490	3.26	2327	3.52	2763	3.37	690	3.34	8270	3.38
DcE	5459	7.16	4716	7.14	5981	7.31	1451	7.02	17,607	7.19

To make things complete, the same procedure was conducted for the specimens that tested negative for Rh D antigen and the results were shown in Table 5. In reality, only seven different Rh phenotypes were identified in the study, in which ce was the most common (56.46%), followed by Cce (30.27%), Ce (6.87%), cEe (4.15%), and CcEe (1.77%). The other two possible Rh phenotypes CcE and CE were not found in our study, however.

**Table 5:** Rh phenotypes frequency in the study of Rh D negative population (*n* = 1470)

Antigen	*ABO*									
	A		B		O		AB		Total	
	Number	Percentage (%)	Number	Percentage (%)	Number	Percentage (%)	Number	Percentage (%)	Number	Percentage (%)
Ce	38	8.19	23	6.87	20	4.02	20	11.56	101	6.87
CcEe	2	0.43	7	2.09	13	2.61	4	2.31	26	1.77
Cce	140	30.17	90	26.87	173	34.74	42	24.28	445	30.27
cE	2	0.43	1	0.30	2	0.40	0	0	5	0.34
cEe	10	2.16	19	5.67	26	5.22	6	3.47	61	4.15
CcE	0	0.00	0	0.00	0	0.00	0	0.00	0	0.00
CEe	1	0.22	0	0.00	1	0.20	0	0.00	2	0.14
CE	0	0.00	0	0.00	0	0.00	0	0.00	0	0.00
ce	271	58.41	195	58.21	263	52.81	101	58.38	830	56.46

### Comparisons with Other Regional Populations in the Literature

In Table 6, we listed the results of the present study and compared them with other available data in terms of Rh antigens in the literature. It was clearly shown that though DCe was found to be the most common Rh phenotype in all the regional populations, the practical frequencies varied much. Indeed, significant differences were noted between the results in Thais and Malaysia and our results. As for the three pieces of research in terms of the Chinese population, the frequency distribution of different Rh phenotypes was in good accordance with each other. One point to be noted was that the frequency distribution of Rh phenotype DCE reported in this study was different from that of the other two available.

**Table 6:** Frequency distributions of Rh antigens and phenotypes in different populations

Antigens and phenotypes	Phenotype frequencies percentage (%)						
	Chinese (this study)	Chinese [25]	Taiwan Chinese [25]	Thais [25]	Chinese in Malaysia [25]	North Indians [25]	Indian [25]
C	89.06	88.00	91.60	95.50	96.00	87.10	84.80
c	53.40	57.50	51.60	34.40	34.50	51.50	17.60
E	45.17	50.40	43.50	32.20	23.00	19.70	56.00
e	92.57	91.20	93.80	96.80	97.50	91.60	99.40
DCe	45.85	41.16	47.80	60.00	61.50	40.95	44.00
DCEe	0.74	0.57	0.90	5.40	3.50	0.32	–
DCE	0.01	0.14	0.00	0.10	0.00	0.00	–
DCce	8.61	7.59	8.20	7.40	15.00	30.91	30.20
DCcEe	33.62	38.87	34.60	22.10	15.00	14.54	10.20
DCcE	0.23	0.64	0.30	0.50	1.00	0.40	–
Dce	0.37	0.36	0.30	0.30	0.00	1.15	2.20
DcEe	3.38	3.65	2.00	1.70	3.00	3.69	6.80
DcE	7.19	7.02	5.90	2.50	1.00	0.78	0.60

### Effectiveness Analysis of the Rh Phenotype Matching Transfusion

**Fig. 2:**
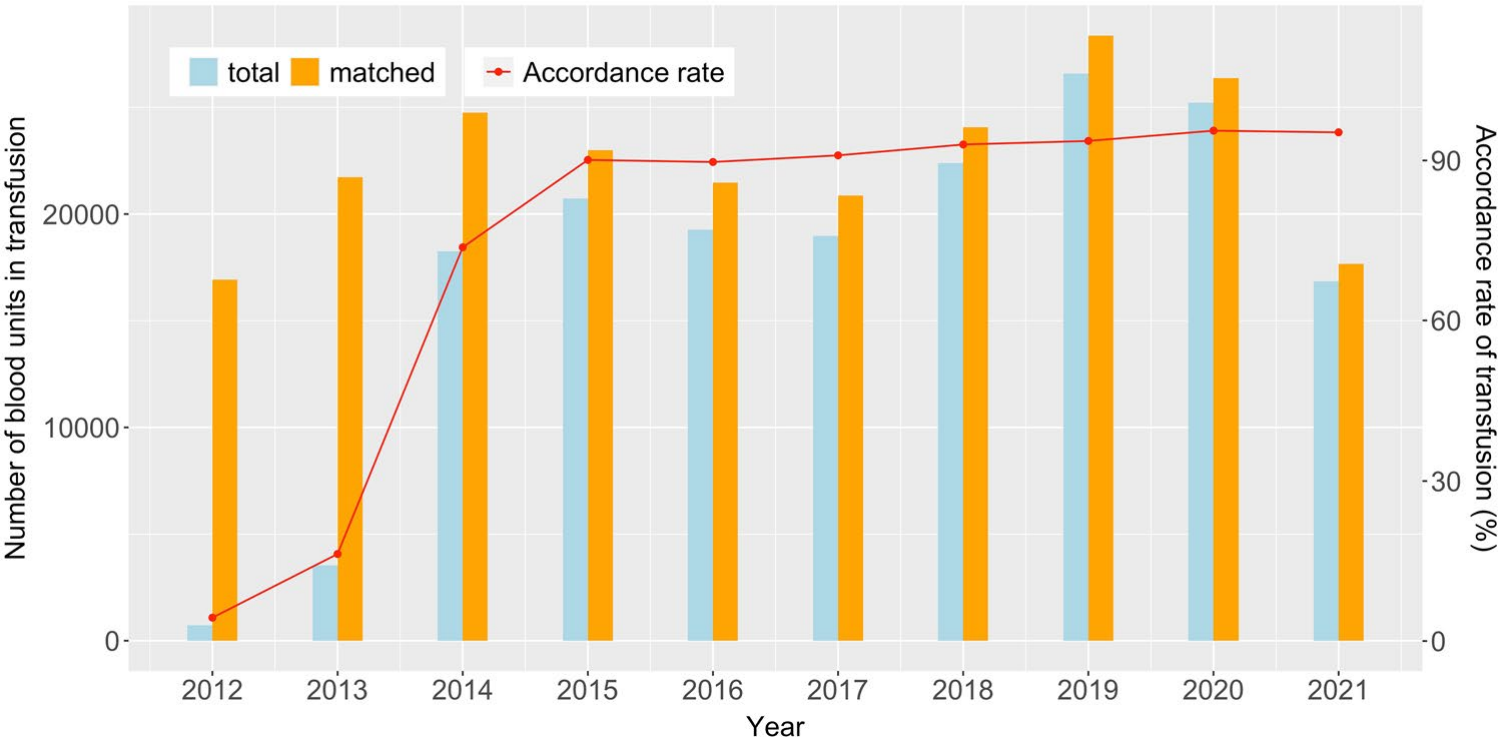
The implementation of Rh antigen phenotype-matched transfusion in our department from 2012 to August 2021: the annual numbers of units of blood transfused (light blue bars), the annual numbers of units of matched blood transfusion (orange bars), and the annual rate of Rh phenotype matching (red line with dots) (color figure online)

As stated in the previous section, our department started to detect the Rh antigens (D, C, E, c, and e) in multi-transfused patients (*n* > 2), and matched them as well as ABO blood group before conducting component blood infusion in 2012. And this practice was generalized to all the patients for blood transfusion in 2014. The resulting rate of Rh antigen phenotype-matched transfusion was shown in Fig. 2, with the numbers of annual units of blood transfusion and the annual units of blood matched. At present, the accordance of Rh phenotype matching has been kept above 95%.

**Fig. 3:**
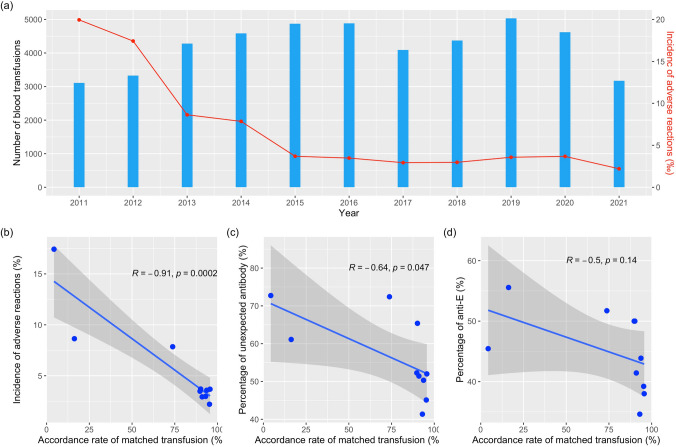
The relations between the accordance rate of Rh phenotype-matched transfusion and various adverse transfusion reactions: the annual change of blood units used in transfusion and the accordance rate of matched blood transfusion (**a**). It is to be noted that in clinical practice, not all the blood transfusions are matched; the correlation between the accordance rate and incidence of adverse transfusion reactions (**b**), the percentage of detected unexpected antibodies (**c**), and the percentage of detected anti-E antibody (**d**)

In this way, the annual number of adverse transfusion reactions recorded decreased from 62 in 2011 to 17 in 2020. Note that in the first 8 months of 2021, 7 transfusion reactions were recorded. With this in mind, we plotted the annual number of blood transfusions and the incidence of adverse reactions in Fig. 3a. It was clearly shown that the incidence of adverse transfusion reactions decreased as the matching of Rh antigens C, E, c, and e was adopted. The incidence rate of adverse transfusion reactions decreased from 19.95‰ in 2011 to 2.21‰ in 2021 (only the first 8 months of the year 2021).

With the remarkable effectiveness of Rh antigen phenotype-matched transfusion, all patients requiring blood transfusion also received antibody screening in pretransfusion testing. In total, 626 patients with unexpected antibodies were found, including 69 autoantibodies and 557 homologous antibodies. Of all the patients with homologous antibodies detected, 323 with Rh blood group system antibodies were identified, among which 82.35% were with anti-E antibodies (266 out of 323). Annual data for the detected unexpected antibodies were shown in Table 7. We again tried to correlate the accordance rate of Rh phenotype-matched transfusion concerning the incidence of adverse transfusion reactions, the percentage of detected unexpected antibodies, and the percentage of detected anti-E antibodies, and plotted the results in Fig. 3b–d, respectively. It was clearly shown that the accordance rate of Rh phenotype-matched transfusion was negatively correlated to the incidence of adverse transfusion reactions with a correlation coefficient of − 0.91. At the same time, moderate correlations were found between the accordance rate of Rh phenotype-matched transfusion and the percentage of detected unexpected antibodies and the percentage of detected anti-E antibodies, though the corresponding *p* values are not showing any statistical evidence. We then asserted with some confidence that the increase in the accordance rate of Rh phenotype-matched transfusion contributed to the decreased incidence of adverse transfusion reactions.

**Table 7:** Distribution of unexpected antibody patients from 2011 to Aug 2021

Year	Number of patients with unexpected antibodies	Number of patients with Rh system antibodies	Number of patients with anti-E antibody	Number of patients with autoantibodies	Detection rate of anti-E antibody (%)
2012	11	8	5	0	45.45
2013	36	22	20	6	55.56
2014	29	21	15	5	51.72
2015	26	17	13	5	50.00
2016	44	23	22	5	50.00
2017	70	36	29	8	41.43
2018	104	43	36	11	34.62
2019	155	78	68	21	43.87
2020	100	52	38	7	38.00
2021	51	23	20	1	39.22
Total	626	323	266	69	42.49

## Discussion

Since the discovery of the ABO blood group system by Landsteiner, many different blood group systems have been identified and reported. According to the ISBT, there are now 345 antigens identified within the 43 blood group systems found. Knowledge about the frequencies of Rh phenotypes can be quite helpful in clinical applications. A donor bank based on this can be established to better provide antigen-negative compatible blood to patients with multiple alloantibodies and multi-transfused patients (e.g. thalassemia). Besides, this can help reduce the risk of alloimmunization caused by the mismatch of antigen phenotypes between blood donors and patients.

Several investigations concerning the Rh phenotypes and genotypes in regional or racial/ethnic populations were available in the literature [10–16]. Based on the clinical operations in our hospital in Zhejiang China since 2012, the current contribution summarized the distributions of Rh phenotypes in the local population and necessitated the detection and matching of Rh phenotypes before blood transfusion. The obtained results were also compared with those available in the literature.

In this study, blood group O was found to be the most common blood group, followed by blood group A. This was consistent with the studies in Europe, America, and South East Asia [10, 11]. However, in some areas such as central Asia and Africa, Blood group B instead of O was the most common blood group system [12, 13].

When it came to the polymorphic Rh blood group system, the frequency of Rh D antigen was as high as 99.4% in the studied population in Zhejiang China, which was significantly different from other racial populations like the white population (85%) and black population (92%) [14].On the contrary, the negative and positive rates of Rh antigen E in the studied population were roughly the same as those shown in the literature. It was confirmed that the detection rate of Rh antigen E was 45.17% in this study. It meant that almost half of the population have different test results in terms of the detection of Rh antigen E. In this regard, we could conclude that the incidence of incompatibility of the Rh E blood group was much higher than that of other Rh blood groups. Hence it was highly possible for patients receiving blood transfusion to produce anti-E antibodies, no matter what amount of blood was transfused. This point was critically important in clinical applications, especially when patients with rare blood groups needed a blood transfusion.

After analyzing the frequencies of Rh antigens (C, E, c, and e) in Rh D positive and negative populations, it was found that in Rh D positive population, Rh antigen e (92.57%) was the most prevalent, followed by Rh antigen C (89.06%). Nonetheless, the prevalence of Rh antigen C (39.05%) was very low in Rh D negative population, while the most prevalent Rh antigen was e (99.66%), followed by Rh antigen c (92.99%). Therefore, it would be clinically difficult to find donors or patients without Rh e antigen [15]. For Rh antigens C and c, it was found that the frequency of Rh antigen c was 53.63%, which was similar to some results presented in the literature [10,11,14,15]. Lower frequencies of Rh antigen c were found in Thais (34.40%) and Malaysia (34.50%) [12,13]. It was found that Rh antigen E was the least common in Rh D positive and negative populations, consistent with previous reports' results [16].

Comparisons of the obtained frequencies of Rh phenotypes in this study with other studies in different ethnic populations were shown in Table 6. The most common Rh phenotype in our study was DCe, accounting for 45.85% of the whole population, while the least common phenotype was DCE constituting only 0.01% of the studied population. This was in good agreement with the results in the literature [10–16]. Among the Rh-negative population, the most common Rh phenotype was ce with a frequency of 56.46%. This was quite different from the result reported by Musa [17].

Variance in the distribution of Rh phenotypes in different populations may lead to different incidences of alloimmunization. Knowledge about the distribution of Rh phenotypes in a specific population could then help formulate clinical blood transfusion guidelines and reduce the significant haemolytic transfusion reactions, especially the delayed haemolytic transfusion reactions (DHTR), difficult crossmatch between blood donors and recipients, decreased survival rate of RBCs after transfusion, and increased blood transfusion requirements. Indeed, considerable alloimmunization was due to Rh blood group antigens. As reported by Dhawan et al. [18] the rate of alloimmunization could be as much as 5.64%, 52.17% of which were due to Rh blood group antibodies (anti-E 17%, anti-D 13%, and anti-C 13%). In a study in Delhi, Agnihotri used the Asian cell panel in a routine pretransfusion test and observed a frequency of alloantibodies 0.8%, of which the most common antibodies detected were related to the Rh blood group (41.6%) [19]. Currently, due to the high immunogenicity of Rh D antigen in around 80% of the Rh D-negative patients, [20] Rh D typing was routinely performed in most countries [21].

However, alloimmunization related to other Rh blood group antigens could not be neglected. In the present study, we screened unexpected antibodies in the pretransfusion test. It was found that of all the 557 homologous antibodies identified, Rh blood group antibodies accounted for 57.99% (323/557), in anti-E accounted for 82.35% (266/323). Noting that Rh blood group system antibodies were produced as a result of immune stimulation, it could be inferred that the high occurrence of anti-E was related to the fact that the positive and negative rates of Rh E antigen were close to each other and that no transfusion with the matched Rh E antigen was adopted in clinical practice (Indeed the clinical practice in our hospital was that only ABO blood group system and Rh D antigen were tested and matched).

Since in the studied region of Zhejiang China, the negative rates of Rh antigens E, C, c, and e were significantly higher than that of Rh antigen D, currently adopted random transfusion based on the pretransfusion matching of Rh antigen D inevitably raised the concern about the alloimmunization related to Rh antigens E, C, c, and e, especially for multi-transfused patients and female patients. A simple solution to the concern is to provide blood with matched Rh antigens in clinical transfusion therapy. For example, for a patient with Rh phenotype DCe, at the first time of receiving a blood transfusion or when anti-E was present, it would be better to provide blood components tested negative for Rh antigens E and c. This was because the immune stimulation related to anti-E probably gave rise to anti-c and anti-cE, while it was generally clinically difficult to detect these antibodies. Similarly, if serological tests indicated the existence of anti-C in a patient with Rh phenotype DcE, the provision of blood component tested negative for Rh antigens C and e were suitable [22–25].

In this sense, we formulated a series of rules for Rh phenotypes matched blood transfusion based on the clinically detected Rh antigens E, C, c, and e in addition to traditional detection of Rh antigen D. Three levels of matching for Rh phenotypes were set up with their priorities from high to low being level I, level II, and level III. In the case of a patient whose Rh phenotypes could not be chosen according to the level I and level II matching rules, the donor blood was chosen with the weakest immunogenicity possible. A detailed list of the formulated rules for different patient phenotypes was shown in the Appendix. Based on these rules, our department started to conduct the Rh phenotypes matching blood transfusion in 2012. With the matching rate of Rh phenotype increased from 4.33% in 2012 to more than 90% after 2017, the incidence of transfusion reactions decreased year by year, from 19.95‰ in 2011 to 2.21‰ in August 2021. Though serological tests were unable to detect genotypes, they still provide a powerful and economical method for the identification of Rh phenotypes in transfusion applications. Later if we could establish the Rh phenotype database of blood donors and perform Rh phenotype testing for each patient before the first blood transfusion, there would be a chance that the incidence of adverse transfusion reactions was reduced and that antigen-negative blood could be provided without delay to save more patients.

## Appendix

See Table

**Table 8:** Rh antigen phenotype matching transfusion

Phenotype	Level I matching	Level II matching
CDe	CDe	Ce
CDEe	CDEe	CDE, CDe, CEe, CE, Ce
CDE	CDE	CCEE
CcDe	CcDe	CDe, cDe, Cce, Ce, ce
CcDEe	CcDEe	Both Rh D positive and Rh D negative all right
CcDE	CcDE	CDE, cDE, CcE, CE, cE
cDe	cDe	ce
cDEe	ccDEe	cDe, cDE, cEe, ce, cE
ccDE	cDE	cE
Ce	Ce	None
CcEe	CcEe	RhD negative all right
Cce	Cce	Ce, ce
cE	cE	None
cEe	cEe	cE, ce
CcE	CcE	CE, cE
CEe	CEe	CE, Ce
CE	CE	None
ce	ce	None

8

In this part, we would like to list the detailed three levels of matching rules adopted in our department. The three levels were named level I, level II, and level III, with the matching priorities from the highest to the lowest.

Level I matching of Rh phenotypes requires that the Rh phenotypes of blood donors and patients are the same. Level II matching of Rh phenotypes refers to the compatibility between the Rh phenotypes of blood donors and patients. The detailed matching rules for these two levels of matching are shown in the following 8. Level III matching of Rh phenotypes aims at reducing the immunogenicity possibly induced by the donor blood.
